# MolFilterGAN: a progressively augmented generative adversarial network for triaging AI-designed molecules

**DOI:** 10.1186/s13321-023-00711-1

**Published:** 2023-04-08

**Authors:** Xiaohong Liu, Wei Zhang, Xiaochu Tong, Feisheng Zhong, Zhaojun Li, Zhaoping Xiong, Jiacheng Xiong, Xiaolong Wu, Zunyun Fu, Xiaoqin Tan, Zhiguo Liu, Sulin Zhang, Hualiang Jiang, Xutong Li, Mingyue Zheng

**Affiliations:** 1 Shanghai Institute for Advanced Immunochemical Studies, and School of Life Science and Technology, ShanghaiTech University, 393 Middle Huaxia Road, Shanghai, 201210 China; 2 Drug Discovery and Design Center, State Key Laboratory of Drug Research, Shanghai Institute of Materia Medica, Chinese Academy of Sciences, 555 Zuchongzhi Road, Shanghai, 201203 China; 3 University of Chinese Academy of Sciences, No. 19A Yuquan Road, Beijing, 100049 China; 4 AlphaMa Inc., No. 108, Yuxin Road, Suzhou Industrial Park, Suzhou, 215128 China; 5 ByteDance AI Lab, No. 1999 Yishan Road, Shanghai, 201103 China; 6 School of Pharmacy, East China University of Science and Technology, 130 Meilong Road, Shanghai, 200237 China; 7 School of Pharmaceutical Science and Technology, Hangzhou Institute for Advanced Study, University of Chinese Academy of Sciences, 310024 Hangzhou, China

**Keywords:** De novo molecule design, Generative models, Deep learning, Virtual screening, Compound quality control

## Abstract

Artificial intelligence (AI)-based molecular design methods, especially deep generative models for generating novel molecule structures, have gratified our imagination to explore unknown chemical space without relying on brute-force exploration. However, whether designed by AI or human experts, the molecules need to be accessibly synthesized and biologically evaluated, and the trial-and-error process remains a resources-intensive endeavor. Therefore, AI-based drug design methods face a major challenge of how to prioritize the molecular structures with potential for subsequent drug development. This study indicates that common filtering approaches based on traditional screening metrics fail to differentiate AI-designed molecules. To address this issue, we propose a novel molecular filtering method, MolFilterGAN, based on a progressively augmented generative adversarial network. Comparative analysis shows that MolFilterGAN outperforms conventional screening approaches based on drug-likeness or synthetic ability metrics. Retrospective analysis of AI-designed discoidin domain receptor 1 (DDR1) inhibitors shows that MolFilterGAN significantly increases the efficiency of molecular triaging. Further evaluation of MolFilterGAN on eight external ligand sets suggests that MolFilterGAN is useful in triaging or enriching bioactive compounds across a wide range of target types. These results highlighted the importance of MolFilterGAN in evaluating molecules integrally and further accelerating molecular discovery especially combined with advanced AI generative models.

**Supplementary Information:**

The online version contains supplementary material available at 10.1186/s13321-023-00711-1.

## Introduction

It has always been the dream of medicinal chemists to design molecules from scratch that meet predefined requirements. However, due to the complexity of drug-target interactions and insufficient understanding of structure–property relationships, it is challenging to find an explicit inverse mapping function to derive chemical structures from the molecular activity or physicochemical properties or absorption, distribution, metabolism, excretion and toxicity (ADMET) properties [1, 2]. Deep generative models such as variational autoencoders (VAEs) [3, 4], generative adversarial networks (GANs) [5, 6], recurrent neural networks (RNNs) [7–10], flow-based models [11, 12], transformer-based models [13, 14], diffusion models [15, 16] and variants or combinations of these models [17–21] have quickly advanced and opened a new path for generating molecules without an explicit inverse mapping function [1, 22]. These models can be easily used to sample novel molecular structures. Moreover, when combined with Bayesian optimization [3, 23], genetic algorithms [24, 25] or reinforcement learning [26–32], generative models are capable of optimizing hits in the desired direction in silico. In the past few years, generative models have been successfully applied in hit discovery and have shown promise in hit-to-lead optimization [19, 29, 33–41].

**Fig. 1:**
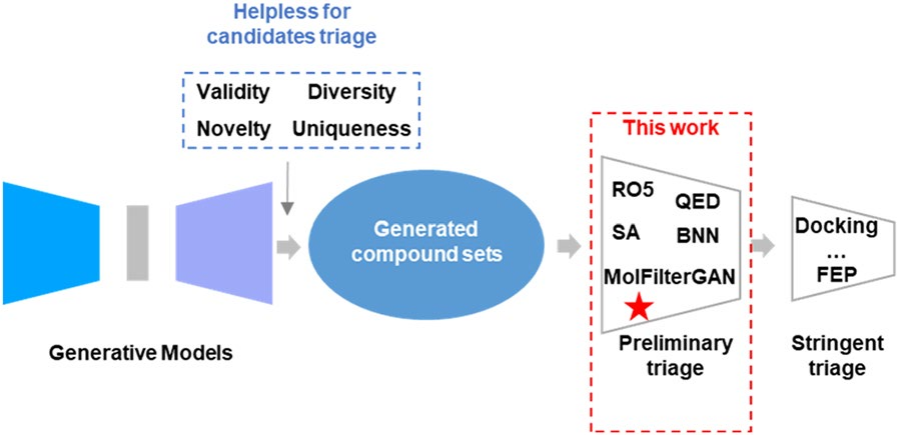
The dilemma of the generative model and the contribution of this work

In the field of generative algorithms, many efforts have been devoted to achieving better performance on related evaluation metrics such as validity (the proportion of chemically valid molecules), uniqueness (the proportion of non-repetitive molecules), novelty (the proportion of unique molecules not included in the training set) or diversity. However, these metrics are not sufficient to characterize the potential of molecules for subsequent development [18–20, 27, 42–44] (see Fig. 1). In addition, considering that the molecular generation process can be easily scaled up, an equally or even more important issue is how to select from the generated molecules for subsequent synthesis and biological evaluation [1, 45–48]. For example, in a report by Zhavoronkov et al., multi-step procedures including many in-house defined filtering methods and expert evaluation by medicinal chemists were adopted in selecting AI designed molecules, which are not readily applicable to other drug design scenes [29].

Many empirical or machine learning-based metrics have been developed for quickly evaluating the potential of molecules. For example, Lipinski summarized the rule-of-five (RO5) from drugs at the time to evaluate the drug-likeness of molecules [49]. Bickerton et al. proposed the quantitative estimate of drug-likeness (QED) by constructing a multivariate nonlinear function from orally administered drugs and known protein ligands (deposited in the Protein Data Bank [50]) to quantify the drug-likeness of molecules [51]. Ertl et al. proposed synthetic accessibility (SA) to quantify the synthesizability of molecules by using a fragment contribution approach, where rarer fragments (as judged by their abundance in the PubChem database) are taken as an indication of lower synthesizability [52]. Lovering et al. proposed Fsp^3^ by counting the proportion of sp^3^ hybridized carbon atoms in total number of carbon atoms to quantify the complexity of spatial structures of molecules [53, 54]. Ivanenkov et al. proposed MCE-18 by counting the presence or proportion of certain structural features (e.g., aromatic or heteroaromatic ring (AR), aliphatic or heteroaliphatic ring (NAR), chiral center (CHIRAL), and spiro point (SPIRO)) to quantify the novelty of molecules [55]. While several studies have used some above metrics to compare the performance of different generative models, how these metrics themselves perform has rarely been discussed in such studies [46, 56].

Recently, AI-based approaches have also been developed for molecule filtering to consider molecular properties implicitly. For example, Hu et al. trained an autoencoder (AE) to classify drug-like molecules (ZINC World Drug) and non-drug-like molecules (ZINC All Purchasable) [57]. Hooshmand et al. [58] and Lee et al. [59] developed self-supervised and unsupervised learning methods to make full use of unlabeled data and predict new drug candidates. Beker et al. extended Hu’s work and improved the discrimination ability by combining several different classifiers like multilayer perceptrons (MLP), graph convolutional neural networks (GCNN) and AE with uncertainty quantification from Bayesian neural networks (BNNs). Though BNN (AE + GCNN), which combines AE and GCNN classifiers, was reported to distinguish drugs from non-drug-like molecules with a 93% accuracy, it failed to recognize common hydrocarbons (e.g., benzene or toluene) as non-drug-like molecules [60]. Overall, all these models are not suitable for all scenarios and were trained and evaluated on disparate datasets. It remains a question how well these metrics will be when they are used for triaging molecules designed by advanced AI methods.

In this study, we first discuss the effectiveness of existing metrics or models on eight benchmark datasets, wherein the molecules are derived from different generated models, common compounds databases, bioactivity databases and approved drug library. Second, we propose MolFilterGAN to distinguish the potential of molecules from different sources and accelerate the virtual screening progress without expert-dependent knowledges. Specifically, the generator tries to generate molecules that the discriminator considers “real” (more like known drugs or bioactive molecules reported), while the discriminator tries to distinguish between “fake” (more like randomly synthesized organic compounds without obvious application purpose) molecules and “real” molecules. After adversarial training, the discrimination logits of final discriminator may serve as a molecule filtering metric for deep generative models. Furthermore, we analyze the effectiveness of the progressively augmentation strategy which means sampling from the produced molecules of the generator of MolFilterGAN at different adversarial training stages to improve the quality of sampling instead of just sampling from a fixed chemical space. In this way, the gradually fine-tuned generator will produce more diverse and balanced negative samples that are increasingly confusing to the discriminator and thus enable the discriminator to gain better discrimination and generalization capability [61, 62].

## Methods

### Data preprocessing

The data cleaning procedures were similar to those used by Hu et al. [57] and the following steps are consistent for all raw data collected: (1) Molecules containing elements beyond H, C, N, O, F, P, S, Cl, Br or I were removed. (2) Molecules containing isotopes were removed. (3) Duplicative molecules were removed. (4) To reduce data bias, molecules with long aliphatic chains (> 4), polyhydroxyl groups (> 10), MW > 750, and atom numbers < 10 were removed. (5) All molecules were transformed to canonical simplified molecular input line entry specifications (SMILESs) with atom chiral information included [63]. (6) Furthermore, a vocabulary was constructed for processing the input SMILES of MolFilterGAN into tokens and those SMILESs containing out-of-vocabulary tokens were removed (for details of the vocabulary, see Additional file 1: Table S1).

### Benchmark datasets

To compare existing molecular filtering metrics, eight different datasets were prepared to represent the chemical space of AI-designed molecules, synthetically accessible molecules, bioactive molecules and approved drugs. Specifically, 10,000 molecules were sampled from each of three advanced generative approaches, including the graph-based genetic algorithm [46, 64] (GA), GENTRL trained with a filtered (molecular weight ranging from 250 to 350, rotatable bonds not greater than 7 and XlogP less than or equal to 3.5) ZINC database [29] (VAE-ZINC-S) and LSTM model trained with the ZINC database [7] (LSTM-ZINC). In addition, we separately sampled 10,000 molecules from ZINC [65] and REAL [66] to represent the general accessible chemical space. Moreover, we sampled 10,000 molecules from ChEMBL [67] (a manually curated validated bioactive compound database) and the Chinese Natural Product Database (CNPD) [68] respectively, which represent the bioactive chemical space. In the end, 748 drug candidates that passed phase III clinical trials were collected from Cortellis to represent the drug chemical space (Cortellis-Drugs, https://clarivate.com/cortellis/, 2020).

### Molecular representation

**Fig. 2:**
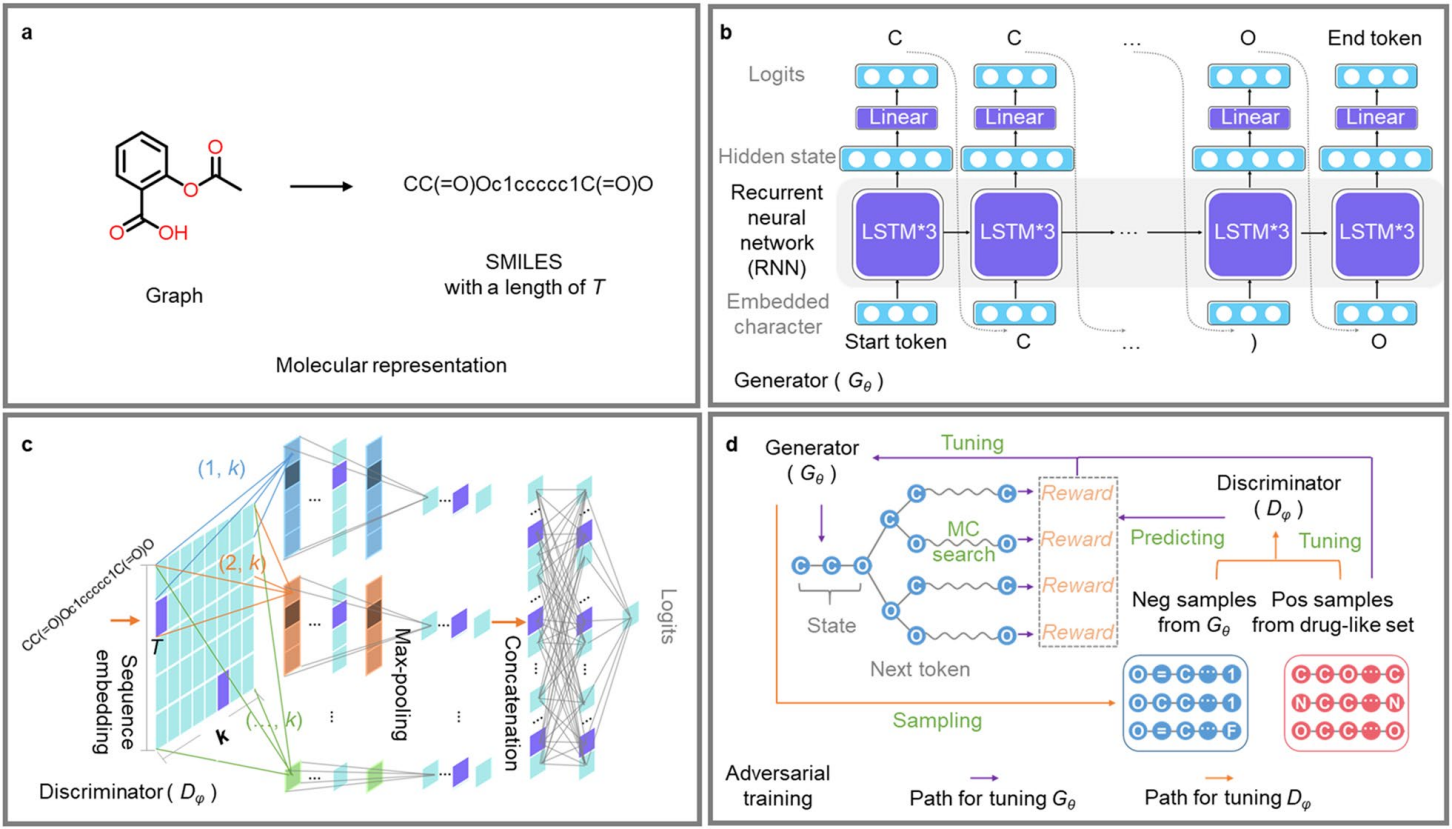
Introduction of MolFilterGAN. **a** Molecular representation. A molecule is represented as a SMILES string with a length of $T$. **b** The generator ${G}_{\theta }$ contains three LSTM cells and one linear layer. Both the input and output of ${G}_{\theta }$ are SMILES strings. **c** The discriminator ${D}_{\varphi }$. The input is a SMILES string, and the output is the probability that the sample belongs to the positive set. The SMILES string is first embedded into a $T\hspace{0.17em}$× *k* matrix, where $T$ is the length of the string and *k* is the size of each embedding vector. Then, multiscale convolution kernels ((1, *k*), (2, *k*), (…, *k*)), max-pooling and a concatenation operation are applied. Finally, a linear layer is used to output the probability. **d** Adversarial training. The generator is tuned by maximizing the rewards predicted by the discriminator. The discriminator is tuned by minimizing the error of discriminating between “fake” samples from the generator (negative set) and “real” samples (positive set)

Generally, molecules are represented as graphs in which atoms are labeled nodes and bonds are edges labeled with the bond order (such as single, double or triple). In the field of natural language processing, the input and output of the model are usually sequences of words or tokens. We therefore employed SMILES, which encodes molecular graphs as human-readable strings. The SMILES grammar describes the molecular structure with characteristics, e.g., c and C for aromatic and aliphatic carbon atoms, O for oxygen atoms, and −, =, and # for single, double, and triple bonds, respectively (see Fig. 2a). In addition, SMILES is, in most cases, tokenized based on a single character. Here, some optimizations were applied according to Olivecrona's work to reduce the generation of invalid SMILES [32], including single atoms represented by multiple characters, such as [C@H], [C@@H], [nH], [C@@], [C@], [S@], [S@@] and [H], which were treated as one token, and Cl and Br were replaced by L and R, respectively. For the generator, both the input and output are SMILES strings. For the discriminator, the input is a SMILES string (molecule), while the output is the probability that the discriminator thinks the string is from the “real” samples (positive set).

### The generative model

The molecule generation problem is denoted as follows. Given a real-world dataset, a *θ* parameterized generative model (${G}_{\theta }$) is trained to produce a sequence (molecule)${W}_{1:T}=\left({w}_{1},\dots ,{w}_{t},\dots ,{w}_{T}\right), {w}_{t}\in V$, where $V$ is the token vocabulary and $T$ is the length of the sequence. This problem can be interpreted from the perspective of reinforcement learning [69]. At time step $t+1$, the state $s$ represents the tokens produced (${W}_{1:t}=\left({w}_{1},\dots ,{w}_{t}\right)$), and action $a$ is the next token to choose (${w}_{t+1}\in V$). Thus, the generation of sequences (molecules) is determined by the policy model ${G}_{\theta }({w}_{t+1}|{W}_{1:t})$. As shown in Fig. 2b, a RNN maps the prior hidden state ${{\varvec{h}}}_{t-1}$ as well as the current input token embedding representation ${{\varvec{x}}}_{t}$ into hidden state ${{\varvec{h}}}_{t}$ at time step $t$ by using the update function $f$ recursively:

1 $${{\varvec{h}}}_{t}=f\left({{\varvec{h}}}_{t-1},{{\varvec{x}}}_{t}\right),$$

Additionally, a softmax layer $z$ maps the hidden states into the output token probability distribution:

2 $$p\left({w}_{t+1}|{w}_{1},\dots ,{w}_{t}\right)=z\left({{\varvec{h}}}_{t}\right)=softmax\left({\varvec{c}}+{\varvec{M}}{{\varvec{h}}}_{t}\right),$$

where ${\varvec{c}}$ is a bias vector and ${\varvec{M}}$ is a weight matrix. In this research, three long-short-term memory (LSTM) cells were used to implement the update function $f$ in Eq. (1) [70]. (For more details, see Additional file 1: Table S2)

### The discriminative model

The discriminative model is shown in Fig. 2c. In this study, a convolutional neural network (CNN) [71] was chosen to train the discriminative model (${D}_{\varphi }$), as it has been successfully applied for many sequence-based molecular classifications [70, 72]. The input embedding representation ${{\varvec{\varepsilon}}}_{1:T}$ of the sequence with a length of T are represented as:

3 $${{\varvec{\varepsilon}}}_{1:T}={{\varvec{x}}}_{1}\oplus \dots \oplus {{\varvec{x}}}_{t}\oplus \dots \oplus {{\varvec{x}}}_{T},$$

where ${{\varvec{x}}}_{t}\in {\mathbb{R}}^{k}$ is a token embedding vector and ⊕ is the concatenation operator for building ${{\varvec{\varepsilon}}}_{1:T}\in {\mathbb{R}}^{T\times k}$. Then, a kernel matrix ${\varvec{\omega}}\in {\mathbb{R}}^{l\times k}$ is used for applying the convolutional operation to a window size of ($l$) words to produce a new feature map ${c}_{i}$:

4 $${c}_{i}=\rho \left({\varvec{\omega}}\otimes {{\varvec{\varepsilon}}}_{i:i+l-1}+b\right),$$

where $\otimes$ defines the summation of element-wise production, $b$ is a bias term and $\rho$ is a nonlinear function. Here, various kernels with different window sizes are used to extract different features. After that, max-pooling and a concatenation operation are applied over the feature maps. Finally, a fully connected layer is used to output the probability that the discriminator thinks the input sequence (molecule) is from the “real” samples (positive set) [70]. (For more details, see Additional file 1: Table S3)

### Adversarial training

The generative model (${G}_{\theta }$) is trained to produce SMILES samples. In contrast, the discriminative model (${D}_{\varphi }$) is trained to distinguish between “real” samples and “fake” samples. As shown in Fig. 2d, ${G}_{\theta }$ is trained to deceive ${D}_{\varphi }$, and ${D}_{\varphi }$ is trained to correctly identify whether samples come from ${G}_{\theta }$ or the positive set. Both models are trained in alternation during adversarial training. Specifically, ${G}_{\theta }$ is trained as an agent in a reinforcement learning context using the REINFORCE algorithm [73]. The agent’s policy is given by ${G}_{\theta }({w}_{t+1}|{W}_{1:t})$, and the objective function ($J(\theta )$) of ${G}_{\theta }({w}_{t+1}|{W}_{1:t})$ is represented as:

5 $$J\left(\theta \right)=\sum_{a\in V}{G}_{\theta }(a|{s}_{t})\cdot Q({s}_{t},a),$$

where ${s}_{t}$ is the state of the agent at step $t$, $a$ is the next action to choose, $V$ is the vocabulary tokens and $Q({s}_{t},a)$ is the action-value function that represents the expected reward of taking action $a$ at state ${s}_{t}$. At step $T-1$, $Q({s}_{T-1}={W}_{1:T-1},a={w}_{T})$ can be predicted by ${D}_{\varphi }({W}_{1:T})$. Since we also want to calculate the action-value for incomplete sequences at intermediate time steps, *N* Monte Carlo searches are applied to policy ${G}_{\theta }$:

6 $${\mathrm{MC}}^{{G}_{\theta }}\left({W}_{1:t};N\right)=\left\{{W}_{1:T}^{1},\dots ,{W}_{1:T}^{n},\dots ,{W}_{1:T}^{N}\right\},$$

where ${W}_{1:t}^{n}$=${W}_{1:t}$ and ${W}_{t+1:T}^{n}$ is sampled by ${G}_{\theta }$. Now action-value becomes:

7 $$Q\left({W}_{1:t},{a}_{t+1}\right)=\left\{\begin{array}{ll}\frac{1}{N}\sum_{n=1}^{N}{D}_{\varphi }\left({\mathrm{MC}}^{{G}_{\theta }}\left({W}_{1:t}^{n};N\right)\right) &\quad t<T-1 \\ {D}_{\varphi }\left({W}_{1:T}^{n}\right),&\quad t=T-1\end{array}\right.$$

An unbiased estimation of the gradient of $J(\theta )$ can be derived as:

8 $${\nabla }_{\theta }J\left(\theta \right)\simeq \frac{1}{T}\sum_{t=1}^{T}{\mathbb{E}}_{{a}_{t+1}\sim {G}_{\theta }\left({a}_{t+1}|{W}_{1:t}\right)} \left[{\nabla }_{\theta }\mathrm{log}{G}_{\theta }\left({a}_{t+1}|{W}_{1:t}\right)\cdot Q\left({W}_{1:t},{a}_{t+1}\right)\right],$$

where expectation ${\mathbb{E}} [\cdot ]$ is approximated by sampling methods. Then, ${G}_{\theta }$ can be updated as:

9 $$\theta \leftarrow \theta +\alpha {\nabla }_{\theta }J\left(\theta \right),$$

where $\alpha$ is the learning rate. Once ${G}_{\theta }$ is updated, ${D}_{\varphi }$ can be tuned as:

10 $$\underset{\varphi }{\mathrm{min}}-{\mathbb{E}}_{W\sim {p}_{real}}\left[\mathrm{log}{D}_{\varphi }\left(W\right)\right]-{\mathbb{E}}_{{W}^{`}\sim {G}_{\theta }}\left[\mathrm{log}(1-{D}_{\varphi }\left({W}^{`}\right)\right]$$

where ${\varvec{W}}$ and ${{\varvec{W}}}^{`}$ are the samples (molecules) from the positive set and negative set (sampled from ${{\varvec{G}}}_{{\varvec{\theta}}}$), respectively [69].

### Details for training MolFilterGAN

To develop MolFilterGAN’s capability to quantify the likelihood that compounds are worthy of further development, a positive set (“real” samples) is needed to allow MolFilterGAN to implicitly learn which kind of molecules are more desirable. Here, the “real” samples were collected from DrugBank [74] (9662), DrugCentral [75] (4053), SuperDRUG2 [76] (3982), CDEK [77] (4421) and Cortellis (25,217, all compounds except those that have passed phase III clinical trials). These compounds from different sources were first cleaned up through the data preprocessing steps described above and then merged to remove duplications as well as those present in the benchmark sets, resulting in a total of 15,955 “real” samples.

Before the adversarial process begins, the initial generator and discriminator of MolFilterGAN need to be trained respectively in advance.

The initial generator was trained with samples from the ZINC [65] library, which is a repository of commercially available small molecules and contains a high proportion of non-drug-like members [60]. A total of 5,000,000 molecules (molecules were first cleaned up with the data preprocessing steps, and those present in the benchmark sets were removed, resulting in a total of 4,338,796 molecules) were randomly sampled from ZINC to make the selected structures as diverse as possible. During training, 100,000 molecules were randomly chosen for monitoring the state of the generator as the validation set, and the remaining ones were used as the training set. The initial generator was trained with a batch size of 512 and a learning rate of 0.0001, and the training process was stopped when the mean loss value on the validation set did not decrease for one epoch to avoid overfitting (see Additional file 1: Fig. S1a).

To train the initial discriminator, the positive set and negative set should be provided. In this research, the above collected 15,955 “real” samples were used as the positive set, and the same amount of samples from the GA model were used as the negative set (all negative samples were not included in benchmark sets). Then, the positive set and negative set were merged and further split into a training set, validation set and internal test set at 8:1:1 to train the initial discriminator. The initial discriminator was trained with a batch size of 128 and a learning rate of 0.0001. The training process was stopped when the mean loss value on the validation set did not decrease for one epoch (see Additional file 1: Fig. S1b).

During the adversarial training process, the generator was tuned with a learning rate of 0.0001. The batch size was set to 64, meaning that an update was about to be made to the generator after every 64 sequences had been generated and scored. In order to gradually increase the task difficulty of the discriminator by progressively augmenting its input or feature space, here, a batch of 64 “real” samples were randomly chosen from 15,955 compounds to fine tune the generator during each update. In this way, the generator can be progressively augmented by the drug-like set, and is able to generate samples that are increasingly confusing to the discriminator and thus enabling the discriminator to have a better discrimination capability. Meanwhile, the discriminator was tuned with the same learning rate of the generator. A batch size of 128 was set, where 64 “fake” samples from the generator and the same number of “real” samples from 15,955 compounds were used to update the discriminator. The training process was stopped when the mean loss value of the discriminator did not decrease for one epoch after stabilization (see Additional file 1: Fig. S1c).

In this study, the Adam optimizer was used to train all models due to its stable and robust performance [78].

### Details of docking

The solvent molecules of the receptor (PDB code: 5FDP) were initially removed, and then the Protein Preparation Wizard Workflow provided in Maestro [79] was used to prepare the 3D structure. The pH was set to 7.0 ± 2.0, and other parameters were set as the default. After that, the grid file was generated by the Receptor Grid Generation Module [79]. The 3D coordinates of ligands were generated using LigPrep [80], and their protonation states were determined at pH 7.0 ± 2.0 with Epik [81]. In addition, ligand structures were desalted, and their tautomers were generated as the default. The resulting conformations were docked to the receptor structure using Glide SP mode [82], and other parameters were set as the default. The conformation with the lowest docking score was kept for analysis.

## Results and discussion

### The comparison between existing molecular filtering approaches and MolFilterGAN on benchmark datasets

**Fig. 3:**
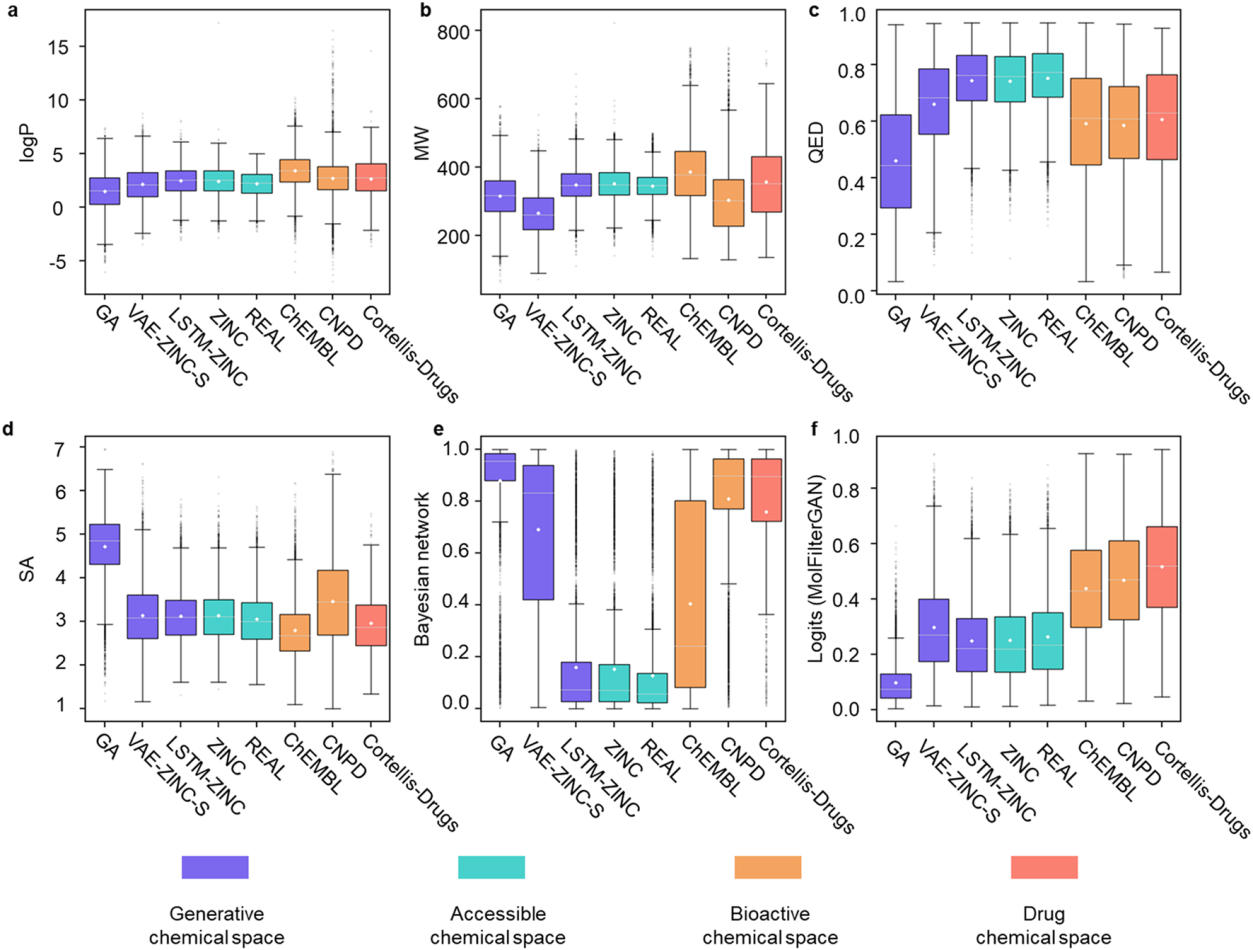
Score distribution of **a** logP (oil/water partition coefficient), **b** MW (molecular weight), **c** QED (0–1, larger, better), **d** SA (1–10, smaller, better), **e** BNN (AE + GCNN) (0–1, larger, better) and **f** logits of MolFilterGAN (0–1, larger, better) on benchmark sets. Molecules sampled from GA (graph-based genetic algorithm) [64], VAE-ZINC-S (GENTRL trained with filtered ZINC database [29]) and LSTM-ZINC (LSTM model trained with ZINC database [7]) are used to represent the generative chemical space. Molecules from ZINC [65] and REAL [66] are used to represent the accessible chemical space. Molecules from ChEMBL [67] and CNPD [68] are used to represent the bioactive chemical space. Molecules from Cortellis-Drugs are used to represent the drug chemical space

In this study, we first tested the scoring distribution of some frequently used molecular filtering approaches or metrics on datasets representing different chemical spaces. RO5 (Lipinski's rule of five) was first evaluated as a simple but extensively utilized rule of thumb for estimating drug-likeness of compounds by medicinal chemists. As shown in Fig. 3a, b and Additional file 1: Fig. S2a–c, most compounds from bioactive chemical space or drug chemical space meet Lipinski's rule of five, however, metrics of RO5 are completely insufficient to prioritize bioactive/drug chemical space (ChEMBL, CNPD and Cortellis-Drugs) from generative chemical space (GA, VAE-ZINC-S and LSTM-ZINC) or general accessible chemical space (ZINC and REAL), which means high false positive rate might occur when RO5 is applied for triaging drug candidates.

Next, we evaluated the most widely used Quantitative Estimate of drug-likeness score (QED [51]) and synthetic accessibility score (SA [52]) in the field of generative models. As shown in Fig. 3c, d, QED and SA cannot prioritize bioactive/drug chemical space either. In contrast, a misleading trend can be observed for QED, where ZINC and REAL were assigned more favorable scores than ChEMBL, CNPD and Cortellis-Drugs, suggesting that it might be counterproductive when they are applied on some commercial libraries for hit screening.

Then, a robust baseline BNN (AE + GCNN), which integrated the predictions of AE and GCNN by retaining predictions with lower uncertainty, was evaluated on the benchmark set (the prediction results of AE and GCNN each on the benchmark datasets are shown in Additional file 1: Fig. S3). As shown in Fig. 3e, the BNN (AE + GCNN) can distinguish the drug chemical space from the general accessible chemical space, and the score distribution of the bioactive library (ChEMBL, CNPD) is also in line with expectations. As benchmarked by Brown et al. in their GuacaMol evaluation framework, a lower proportion of high-quality molecules was found among the samples generated by generative models than those sampled from ChEMBL [46]. Unfortunately, the BNN (AE + GCNN) incorrectly assigned high scores for the generative libraries GA and VAE-ZINC-S. The results above indicate that BNN (AE + GCNN) may be helpful in HTS (High throughput screening) or vHTS (virtual high throughput screening), however, high false positive rate might also occur when it is applied to generative models. Some more metrics were also tested (FSP^3^ and MCE-18, details see Additional file 1: Fig. S2), but none of these frequently used metrics is appropriate for filtering molecules from deep generative models.

The established MolFilterGAN was then evaluated on the same benchmark datasets representing different chemical spaces. As shown in Fig. 3f, MolFilterGAN can distinguish drug or bioactive molecules from those of the general accessible chemical space well. In addition, MolFilterGAN assigns lower scores to VAE-ZINC-S or GA than ChEMBL, which is consistent with the results from Brown et al. [46]. The above results indicate that quite a lot of low-quality generative compounds can be filtered out by MolFilterGAN and the problem of high false positive rate can be alleviated to a large extent. Moreover, we investigated the impact of the percentage of labeled data in positive class (Additional file 1: Fig. S4 and Additional file 1: Table S4), the results show that the percentage of labeled data in positive class can affect MolFilterGAN's ability to discriminate positive samples but has little effect on its ability to discriminate negative samples. Overall, the results suggest that MolFilterGAN shows better performance in discriminating compounds from different sources than existing molecular filtering approaches, therefore it is more adapted to evaluate the molecules benefit from the robust discrimination capability.

### The progressively augmented sampling method makes MolFilterGAN stand out

Both BNN (AE + GCNN) and MolFilterGAN try to train a model to discriminate molecules from different resources. As discussed by Beker et al. [60], the BNN (AE + GCNN) is limited by the unbalanced representation of different molecular types/features in the negative dataset, and we argue that the improvement in MolFilterGAN might be attributed to progressive augmentation training, which makes the negative data more diverse and balanced.

**Fig. 4:**
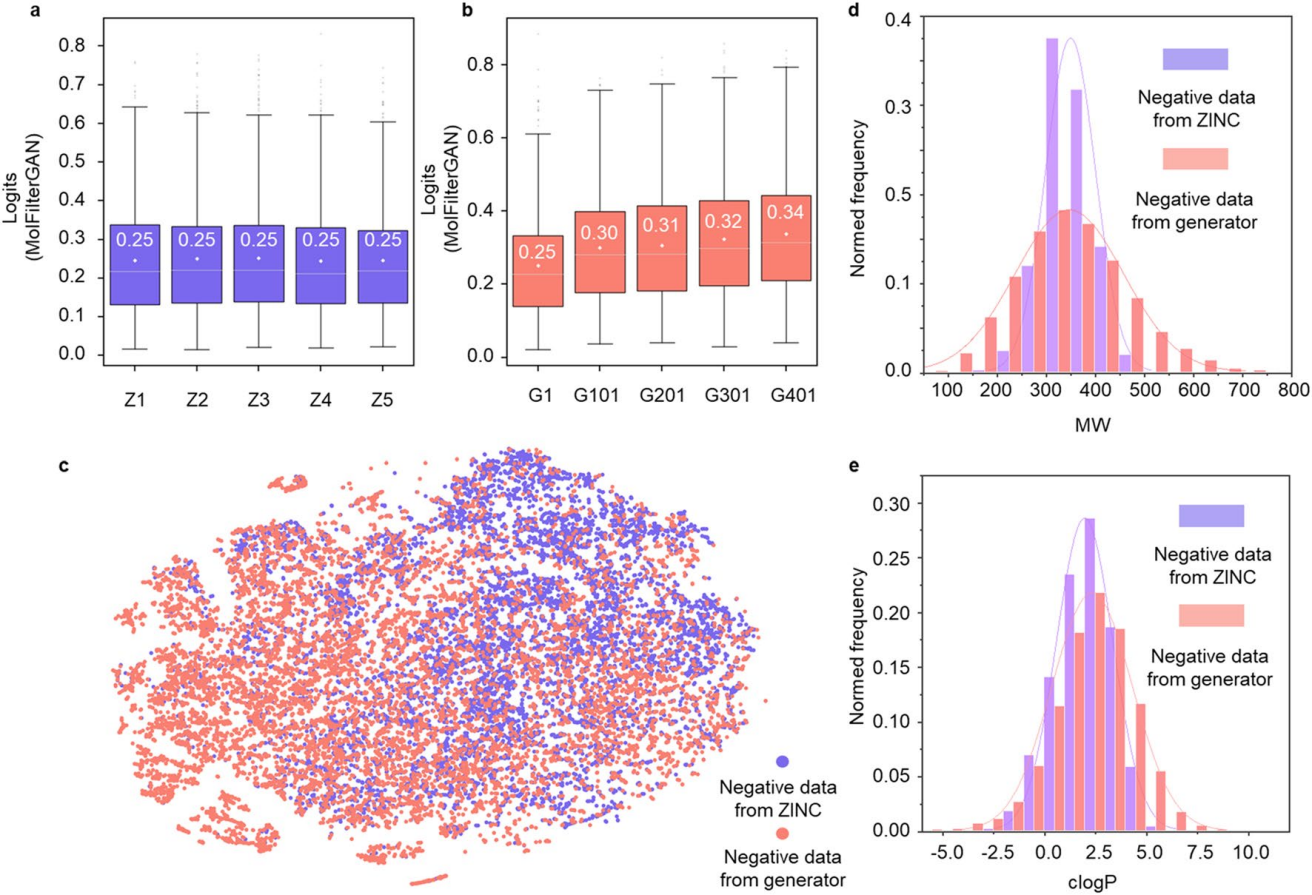
The comparison between a random sampling method and the progressively augmented sampling method. **a** MolFilterGAN scoring distribution for molecules repeatedly sampled from ZINC. **b** MolFilterGAN scoring distribution for molecules sampled from the generators at different training stages. Five compound sets (Z1, Z2, Z3, Z4, Z5) were constructed by repeatedly sampling 1000 molecules from ZINC while the other five sets (G1, G101, G201, G301 and G401) were constructed by sampling compounds from the generators at steps 1, step 101, step 201, step 301 and step 401 respectively. **c** T-SNE plot, **d** molecular weight distribution and **e** clogP distribution for 10,000 molecules sampled by above two methods. T-SNE plot was made by DataWarrior with default settings [83]

A simulation study was carried out to compare these two sampling methods. In detail, 1000 molecules were randomly sampled from ZINC and the process was repeated five times (named from Z1 to Z5) while the same amount of molecules were separately sampled from the generator at five stages (G1, G101, G201, G301 and G401). As shown in Fig. 4a, MolFilterGAN scoring distributions of five sets of molecules repeatedly sampled ZINC are about the same, which means that diversity and representativeness of compounds in the negative set cannot be guaranteed by just including more ZINC data. Meanwhile, we found that MolFilterGAN scoring distribution of molecules sampled from the initial generator (step = 1) is also similar to those sampled from ZINC (see Fig. 4b), which indicates that molecules sampled from the generator at this stage are “ZINC-like”. However, as the training progresses, MolFilterGAN scores of molecules from the generator improves, suggesting that gradually fine-tuned generator is able to produce diverse “fake” samples that are increasingly confusing (more challenging) to the discriminator. To illustrate, we sampled 10,000 molecules from ZINC and the generator at different stages and placed them together in a t-SNE plot. As shown in Fig. 4c, molecules from the generators spread in a wider space compared with those from ZINC, which means that negative data produced by augmented generators are more diverse than that randomly sampled from ZINC.

In addition, we also analyzed the distributions of molecular weight, clogP as well as the number of hydrogen bond acceptors, hydrogen bond donors and rotatable bonds for molecules sampled by above two methods. As shown in Fig. 4d, e and Additional file 1: Fig. S5a–c, molecular weights of ZINC molecules are densely distributed between 200 and 450, and the molecular weight between 250 and 400 accounted for more than 90% of all molecules. In contrast, molecular weights of the molecules generated by MolFilterGAN are widely distributed between 50 and 800. Similarly, the distributions of clogP as well as the number of hydrogen bond acceptors, hydrogen bond donors and rotatable bonds also supports that the negative data for training MolFilterGAN are more diverse and balanced. A given method’s accuracy may vary quite perceptibly depending on the choice of the negative set of “non-drugs” [60].

Here, we show that the progressively fine-tuned generator is able to produce diverse and balanced negative samples that are increasingly confusing to the discriminator, and consequently, the discrimination and generalization capability of MolFilterGAN have been enhanced.

### MolFilterGAN increases the efficiency of filtering generative molecules

**Fig. 5:**
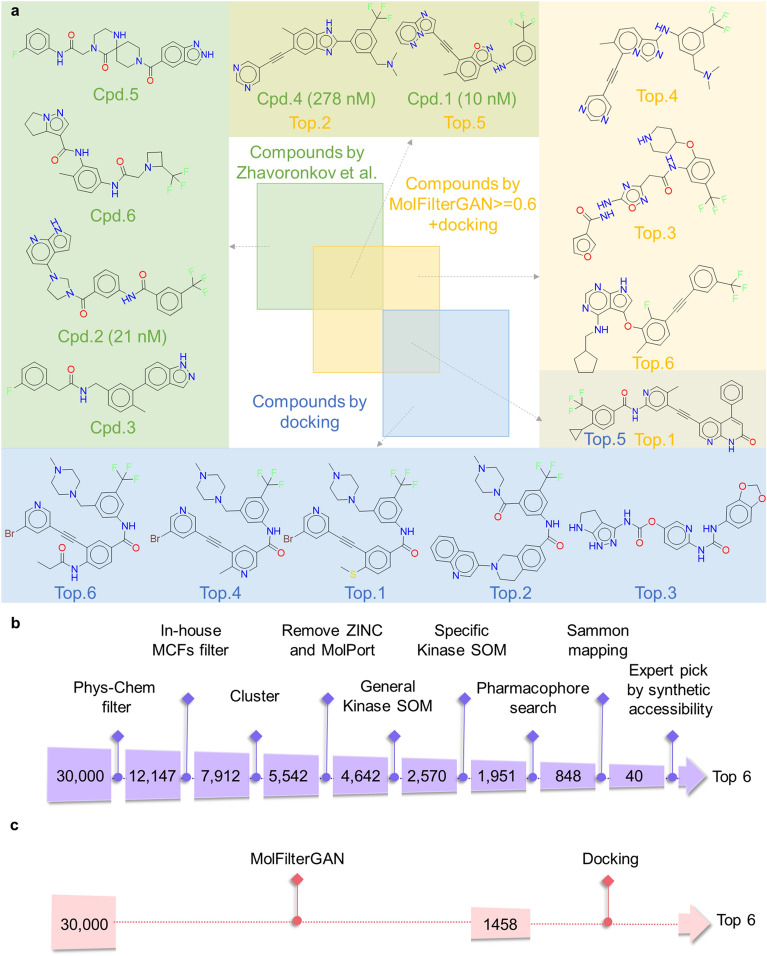
Comparison of the molecular filtering procedures and results on the same set of 30,000 GENTRL-generated compounds reported by Zhavoronkov et al. **a** Venn diagram showing the top-ranked six compounds selected by Zhavoronkov et al. (light green square), docking (light blue square), and MolFilterGAN + docking (light gold square). Flowcharts comparing the molecular filtering procedures of **b** Zhavoronkov et al. and **c** MolFilterGAN + docking

MolFilterGAN was then examined in a real-world case study. Zhavoronkov et al. developed the AI model GENTRL to design and screen discoidin domain receptor 1 (DDR1) inhibitors [29]. Out of 30,000 compounds generated by GENTRL, a variety of in-house filtering approaches were combined with human expert visual inspection to triage the compounds, leading to 6 selected compounds for the subsequent synthesis and biological evaluation. Among them, 3 compounds showed IC_50_ values below 1 µM, and the best compound (cpd.1) showed an IC_50_ of 10 nM. To evaluate the practical usage of MolFilterGAN, a retrospective analysis was performed using MolFilterGAN to filter the same set of 30,000 GENTRL-generated compounds. Here, only MolFilterGAN and conventional structure-based docking were used. As shown in Fig. 5a, none of the 3 active compounds could be ranked within the top 6 by using molecular docking scores alone. Interestingly, when combined with MolFilterGAN, the “true” active cpd.1 and cpd.4 can be successfully retrieved within the top 6. The results suggest that MolFilterGAN can be used as a useful filtering approach for de novo designed molecules. By only using MolFilterGAN and docking, the complicated procedure used by Zhavoronkov et al. can be significantly simplified, as shown in Fig. 5b, c.

### MolFilterGAN is useful in triaging bioactive molecules across a wide range of target types

**Fig. 6:**
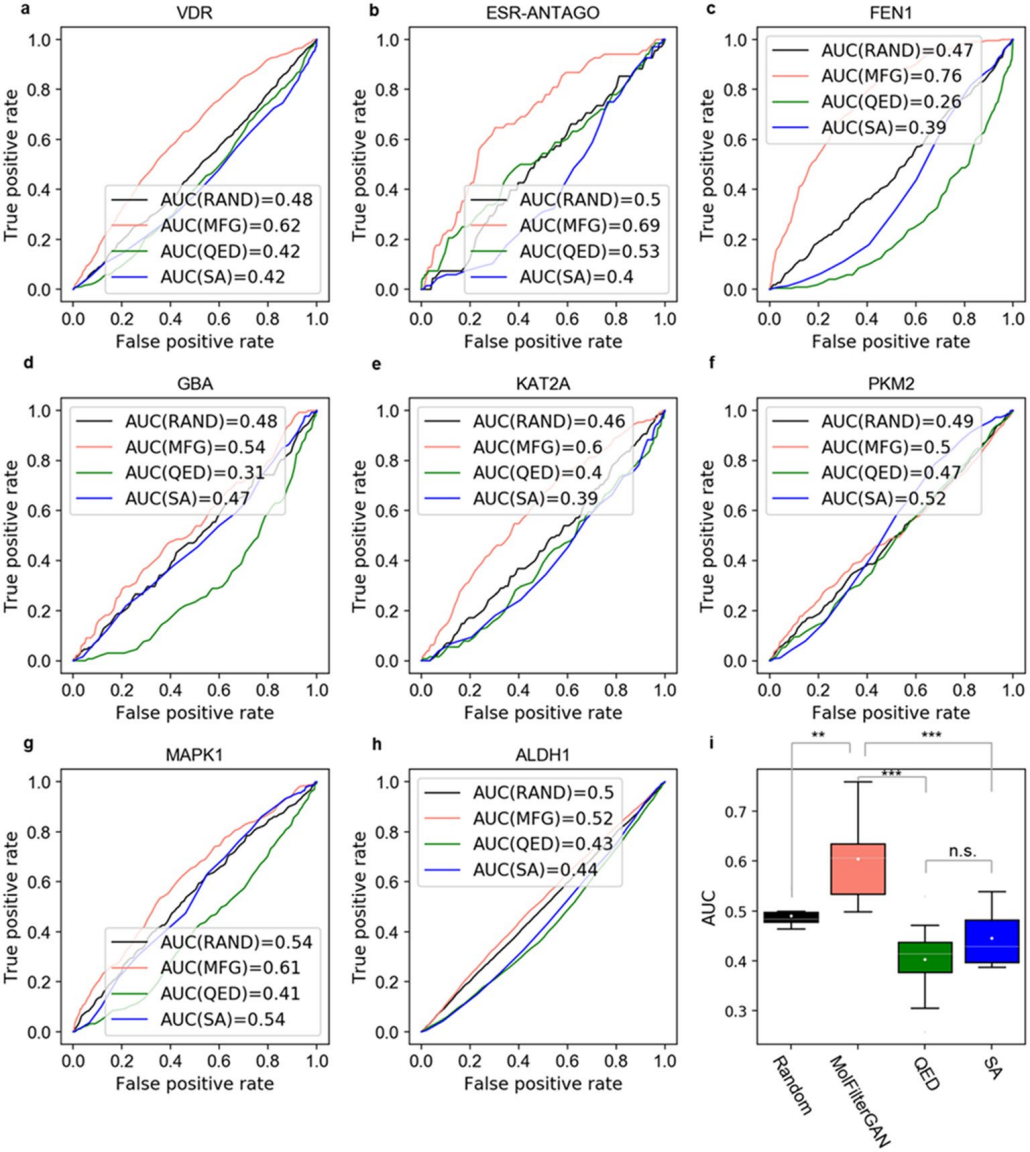
Evaluation of MolFilterGAN, QED and SA on HTS dataset LIT-PCBA. AUC scores for a random guess (RAND), QED, SA and MolFilterGAN (MFG) on 8 target sets, including, **a** VDR, **b** ESR-ANTAGO, **c** FEN1, **d** GBA, **e** KAT2A, **f** PKM2, **g** MAPK1, and **h** ALDH1. The random method, QED, SA and MolFilterGAN are represented as solid black, green, blue and salmon lines respectively. **i** AUC score distribution for the random guess, QED, SA and MolFilterGAN on all target sets. For the random guess, a value of 0 or 1 from a uniform distribution was assigned for each molecule. *p < 0.05, **p < 0.01, ***p < 0.001, and *ns* not significant. A statistical analysis was performed by one-tailed Student’s *t*-test

In addition to the case study of DDR1, MolFilterGAN was further evaluated on LIT-PCBA [84], which is a high-throughput screening (HTS) bioassay dataset where all active and inactive ligands relating to each target were experimentally confirmed. Since the number of active compounds of each target varies greatly, only those targets containing more than 100 active compounds were included, resulting in a test set with 8 targets (VDR, ESR-ANTAGO, FEN1, GBA, KAT2A, PKM2, MAPK1 and ALDH1). For each target, the ligand set was preprocessed as described in data preprocessing section before evaluation. Here QED and SA were used for comparison. In addition, a random prediction model was also benchmarked, where a value of 0 or 1 from a uniform distribution was assigned to each compound. The area under the receiver operating characteristic curve (AUC) score was used to evaluate their performance. Intriguingly, as shown in Fig. 6a–i, the AUC scores of QED and SA were lower than those of a random guess on almost all target sets, which means that QED and SA might deteriorate hit triage when they are applied as filtering metrics. Considering that SA is an indicator of synthesis difficulty, it may cause a higher proportion of simple molecules to be retained when filtering chemical library (see Additional file 1: Fig. S6), thereby reducing the positive rate (i.e. the possibility of molecules possessing biological activity). This may explain why SA is even inferior to random picking. QED is an indicator of drug-likeness of compounds and has been widely used in studies of deep generative models. However, QED was designed to distinguish orally administered drugs from known protein ligands (deposited in the Protein Data Bank) [51]. It means that bioactive compounds or ligands with properties similar to those in the Protein Data Bank are considered negatives, and hence cannot be prioritized. All these results suggest that QED and SA should be used with caution especially when our goal is to find hits during early stage of drug discovery, as these metrics tend to reduce the enrichment of hits. In contrast, the AUC scores of MolFilterGAN were obviously higher than those of the random method on six target sets and were comparable to those of the random method on two target sets, suggesting that MolFilterGAN is indeed useful in triaging active hits across a wide range of target types. Here, the goal of model training is not to discriminate between active and inactive compounds on a specific target, so it is expected that the model did not show greater than random guessing ability on a specific target in this test. However, as shown in Fig. 6, we observed that MolFilterGAN shows a certain discrimination ability on most targets. It is an intriguing result. Since there have been many reports that discriminators from GANs can be used as successful feature extractors [61, 62, 85, 86], our results suggest that MolFilterGAN may have learned the hidden features encoding whether chemicals have structures related to fortuitous biological activity. Moreover, considering that none of the molecular target information was included when training MolFilterGAN, there is few restrictions for the utilization of MolFilterGAN and it is possible to incorporate other orthogonal methods such as docking and binding affinity prediction models.

## Conclusions

Currently, AI-based molecular design methods, such as deep generative models, have demonstrated powerful chemical space exploration capability and promising prospects for new drug discovery. However, these methods face a major challenge in prioritizing molecular structures with potential for subsequent drug development from the extremely huge chemical space. In this study, we first analyzed the effectiveness of some frequently used molecular filtering metrics (RO5, QED, SA and et al.), and strong AI-based models [AE, GCNN and BNN (AE + GCNN)] on datasets representing the generative chemical space, accessible chemical space, bioactive space and drug space. The results show that none of these methods is adequate to distinguish molecules from different sources. Second, based on a generative adversarial network, we developed a novel molecular filtering approach, MolFilterGAN, to address this issue. By expanding the size of the drug-like set and using a progressive augmentation strategy, MolFilterGAN has been fine-tuned to distinguish between bioactive/drug molecules and those from the generative chemical space. Third, we examined the validity of MolFilterGAN by a retrospective analysis of AI-designed DDR1 inhibitors. The results show that MolFilterGAN can significantly increase the efficiency in picking out bioactive compounds from generative molecules. Finally, we evaluated MolFilterGAN on an HTS bioassay dataset where all active and inactive ligands were experimentally confirmed. The results suggest that MolFilterGAN is helpful in triaging bioactive compounds across a wide range of target types. Overall, MolFilterGAN can be used as a practical tool for triaging potential molecules thereby improving the hit rate of active compounds, and the research is expected to accelerate drug discovery by filtering the AI-generated molecules and reduce the heavy reliance on manual evaluation by medicinal chemists in current real-world applications.

## Supplementary Information

**Additional file 1. Fig. S1**: Training details of MolFilterGAN. The mean loss value and epoch curve of **a** training the initial generator, **b** training the initial discriminator, **c** training the discriminator when the generator was tuned with a drug-like set. **Fig. S2**: Score distribution of **a** HBD (Hydrogen bond donors), **b** HBA (Hydrogen bond acceptors), **c** RB (Rotatable bonds), **d** Fsp3 (1–10, smaller, better), and **e** MCE-18 (0–1, larger, better). Fsp3 and MCE-18 are used to characterize the drug-likeness and novelty of compounds. **Fig. S3**: Score distribution of AE (**A**) and GCNN (**B**) on benchmark datasets. **Fig. S4**: Score distribution of MolFilterGAN on benchmark datasets when the percentage of labeled data in positive set are **A** 1%, **B** %5, **C** 10%, **D** 30%, **E** 50%, and **F** 70%. **Fig. S5**: Comparison of the number of **a** hydrogen bond acceptors, **b** hydrogen bond donors and **c** rotatable bonds between the negative data sampled from ZINC and those sampled from the generator at different stages. **Fig. S6**: Some molecules with low SA scores. SA values can range between one (easy to synthesis) and ten (hard to synthesis). **Table S1**: The token vocabulary of MolFilterGAN. **Table S2**: The detail parameters for the generator of MolFilterGAN. **Table S3**: The detail parameters for the discriminator of MolFilterGAN. The vocabulary size, embedding size and dropout are the same as those of the generator. **Table S4**: The relationship between structural diversity and the percentage of labeled data in positive class.

## Data Availability

The token vocabulary and detail parameters of MolFilterGAN are available in the supplementary material. The source code and related datasets are provided for academic use: https://github.com/MolFilterGAN/MolFilterGAN.

## Abbreviations

ADMET: Absorption, distribution, metabolism, excretion and toxicity
AE: AutoEncoder
AI: Artificial intelligence
AR: Aromatic or heteroaromatic ring
AUC: Area under the receiver operating characteristic curve
BNNs: Bayesian neural networks
CHIRAL: Chiral center
CNN: Convolutional neural network
CNPD: Chinese Natural Product Database
DDR1: Discoidin domain receptor 1
MLP: Multilayer perceptrons
GA: Genetic algorithm
GANs: Generative adversarial networks
GCNN: Graph convolutional neural networks
HTS: High throughput screening
LSTM: Long-short-term memory
MW: Molecular weight
NAR: Aliphatic or heteroaliphatic ring
QED: Quantitative estimate of drug-likeness
RNNs: Recurrent neural networks
RO5: Rule-of-five
SA: Synthetic accessibility
SMILESs: Simplified molecular input line entry specifications
SPIRO: Spiro point
VAEs: Variational autoencoders
vHTS: Virtual high throughput screening
