# Supplementary material for: MolFilterGAN: a progressively augmented generative adversarial network for triaging AI-designed molecules
Source: J Cheminform. 2023 Apr 8;15:42. doi: 10.1186/s13321-023-00711-1 (PMC10082991; doi:10.1186/s13321-023-00711-1)
Supplement: Supplementary file 1 — Additional file 1. Fig. S1: Training details of MolFilterGAN. The mean loss value and epoch curve of a training the initial generator, b training the initial discriminator, c training the discriminator when the generator was tuned with a drug-like set. Fig. S2: Score distribution of a HBD (Hydrogen bond donors), b HBA (Hydrogen bond acceptors), c RB (Rotatable bonds), d Fsp3 (1–10, smaller, better), and e MCE-18 (0–1, larger, better). Fsp3 and MCE-18 are used to characterize the drug-likeness and novelty of compounds. Fig. S3: Score distribution of AE (A) and GCNN (B) on benchmark datasets. Fig. S4: Score distribution of MolFilterGAN on benchmark datasets when the percentage of labeled data in positive set are A 1%, B %5, C 10%, D 30%, E 50%, and F 70%. Fig. S5: Comparison of the number of a hydrogen bond acceptors, b hydrogen bond donors and c rotatable bonds between the negative data sampled from ZINC and those sampled from the generator at different stages. Fig. S6: Some molecules with low SA scores. SA values can range between one (easy to synthesis) and ten (hard to synthesis). Table S1: The token vocabulary of MolFilterGAN. Table S2: The detail parameters for the generator of MolFilterGAN. Table S3: The detail parameters for the discriminator of MolFilterGAN. The vocabulary size, embedding size and dropout are the same as those of the generator. Table S4: The relationship between structural diversity and the percentage of labeled data in positive class. [file 13321_2023_711_MOESM1_ESM.docx]

**MolFilterGAN: A Progressively Augmented Generative Adversarial Network for Triaging AI-designed Molecules**

Xiaohong Liu^1,2,3,4†^, Wei Zhang^2,3†^, Xiaochu Tong^2,3^, Feisheng Zhong^2,3^, Zhaojun Li^4^, Zhaoping Xiong^1,2,3^, Jiacheng Xiong^2,3^, Xiaolong Wu^2,6^, Zunyun Fu^2^, Xiaoqin Tan^2,3,5^, Zhiguo Liu^4^, Sulin Zhang^2,3^, Hualiang Jiang^1,2,3^, Xutong Li^2,3*^and Mingyue Zheng^2,3*^

^1^ Shanghai Institute for Advanced Immunochemical Studies, and School of Life Science and Technology, ShanghaiTech University, 393 Middle Huaxia Road, Shanghai 201210, China

^2^ Drug Discovery and Design Center, State Key Laboratory of Drug Research, Shanghai Institute of Materia Medica, Chinese Academy of Sciences, 555 Zuchongzhi Road, Shanghai 201203, China

^3^ University of Chinese Academy of Sciences, No.19A Yuquan Road, Beijing 100049, China

^4^ AlphaMa Inc., No.108, Yuxin Road, Suzhou Industrial Park, Suzhou 215128, China

^5^ ByteDance AI Lab, No.1999 Yishan Road, Shanghai 201103, China

^6^ School of Pharmacy, East China University of Science and Technology, 130 Meilong Road, Shanghai 200237, China

^†^ Xiaohong Liu and Wei Zhang contributed equally to this work

^*^ Correspondence: myzheng@simm.ac.cn; lixutong@simm.ac.cn

CONTENTS

[Figures 3](#_Toc127886153)

[Figure S1, Training details of MolFilterGAN. 3](#_Toc127886154)

[Figure S2, Score distribution of more metrics on benchmark sets. 4](#_Toc127886155)

[Figure S3, Score distribution of AE and GCNN on benchmark datasets. 4](#_Toc127886156)

[Figure S4, Score distribution of MolFilterGAN on benchmark datasets when the percentage of labeled data in positive set changes. 5](#_Toc127886157)

[Figure S5, The comparison between two sampling methods. 6](#_Toc127886158)

[Figure S6. Some molecules with low SA scores. 6](#_Toc127886159)

[Tables 7](#_Toc127886160)

[Table S1, Details of the token vocabulary. 7](#_Toc127886161)

[Table S2, The parameters for the generator. 7](#_Toc127886162)

[Table S3, The parameters for the discriminator. 8](#_Toc127886163)

[Table S4, The impact of the percentage of labeled data in positive class. 9](#_Toc127886164)

# Figures

## Figure S1, Training details of MolFilterGAN.


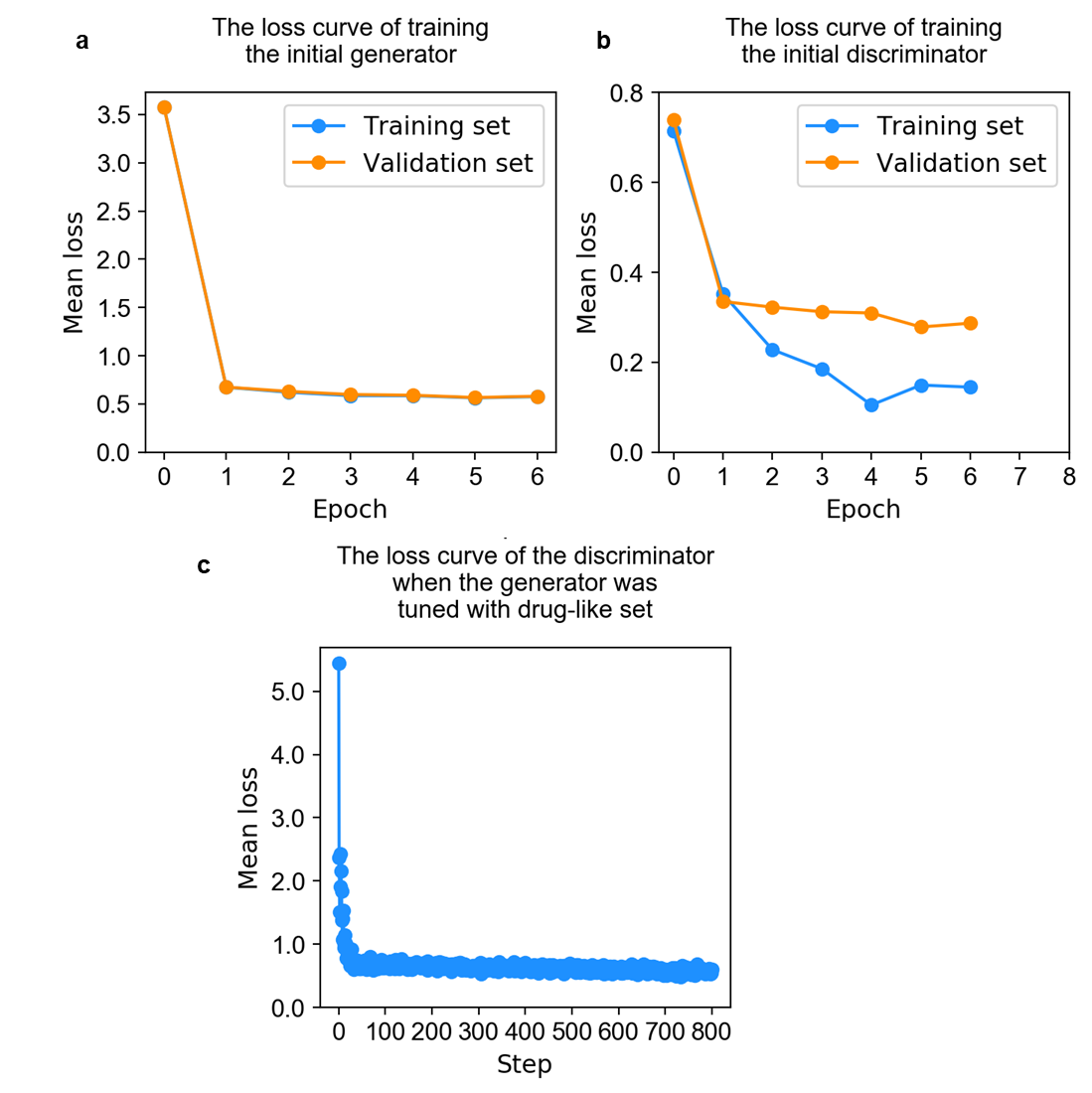


**Figure S1**. Training details of MolFilterGAN. The mean loss value and epoch curve of **a**) training the initial generator, **b**) training the initial discriminator, **c**) training the discriminator when the generator was tuned with a drug-like set.

## Figure S2, Score distribution of more metrics on benchmark sets.


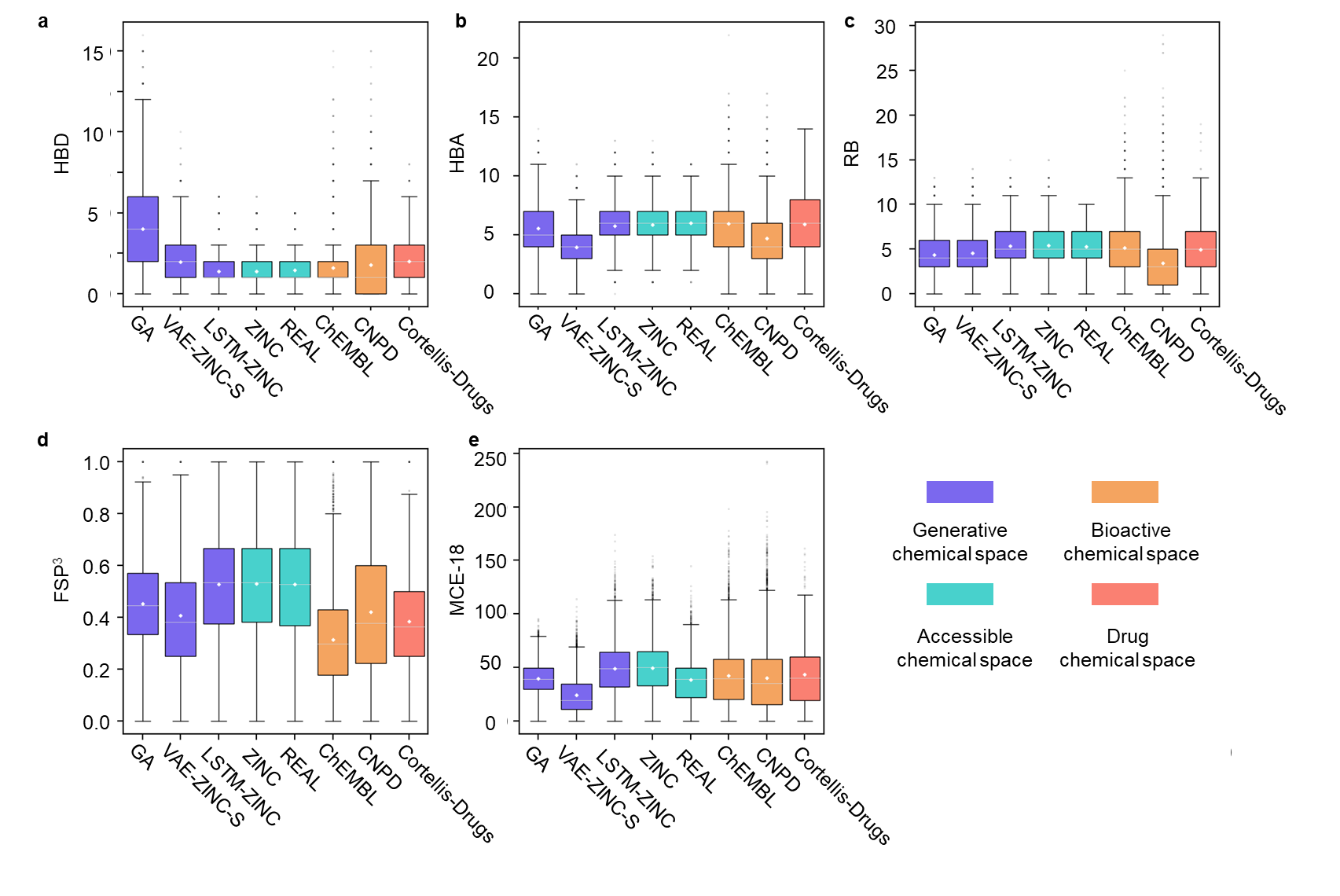


**Figure S2**. Score distribution of (a) HBD (Hydrogen bond donors), (b) HBA (Hydrogen bond acceptors) (c) RB (Rotatable bonds), (d) Fsp^3^ (1~10, smaller, better), and (e) MCE-18(0~, larger, better). Fsp^3^ and MCE-18 are used to characterize the drug-likeness and novelty of compounds.

## Figure S3, Score distribution of AE and GCNN on benchmark datasets.

**
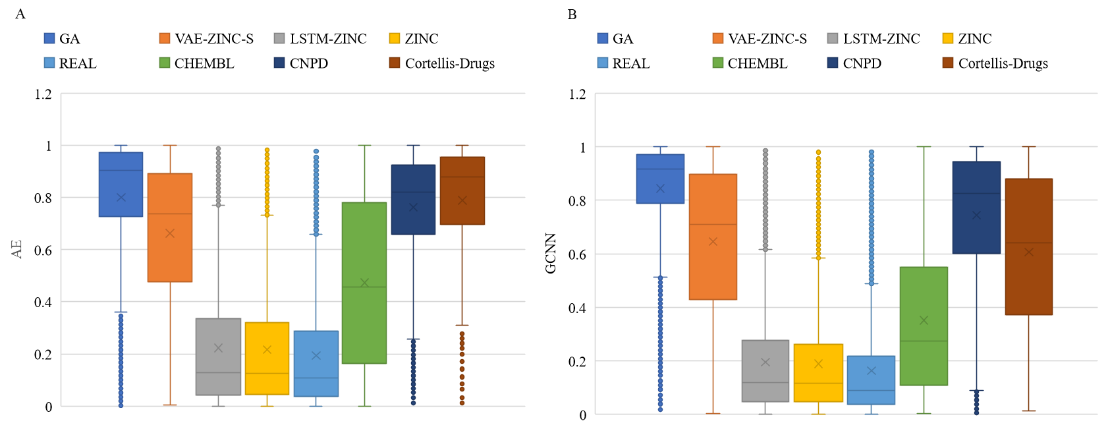
**

**Figure S3**. Score distribution of AE (A) and GCNN (B) on benchmark datasets.

## Figure S4, Score distribution of MolFilterGAN on benchmark datasets when the percentage of labeled data in positive set changes.

**
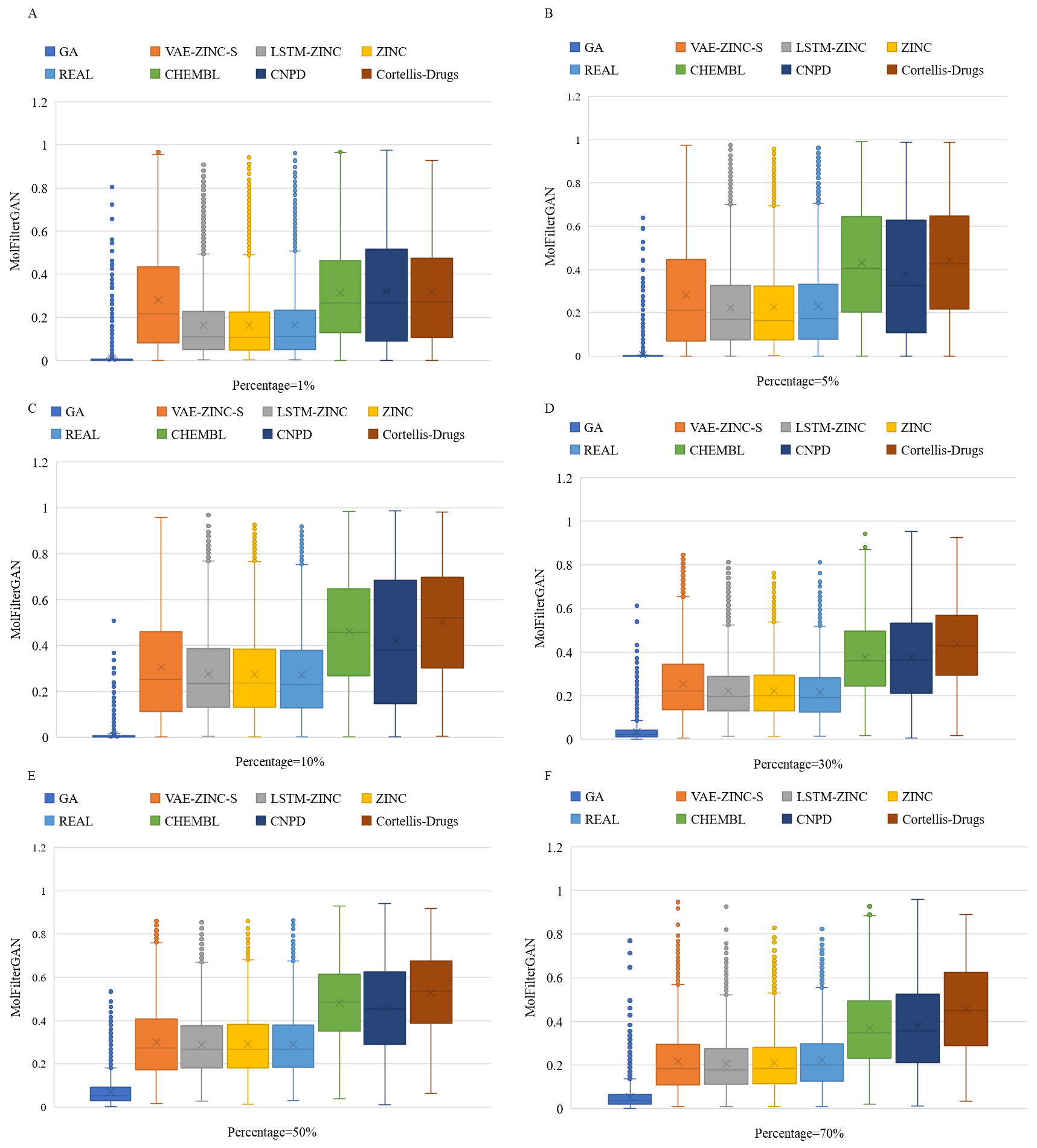
**

**Figure S4**. Score distribution of MolFilterGAN on benchmark datasets when the percentage of labeled data in positive set are (A) 1%, (B) %5, (C) 10%, (D)30%, (E)50%, and (F)70%.

## Figure S5, The comparison between two sampling methods.


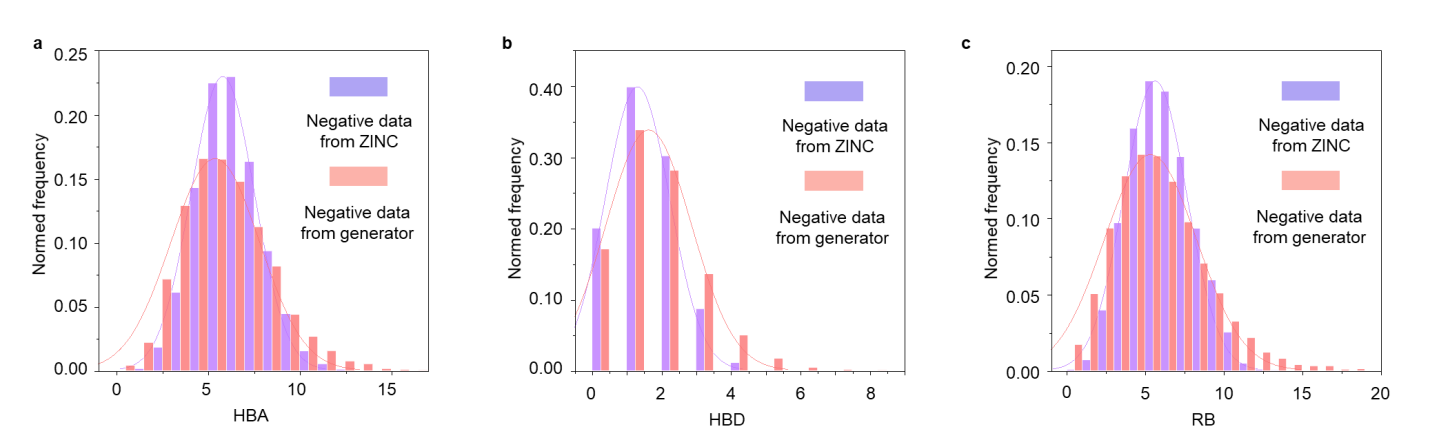


**Figure S5**. Comparison of the number of **a**) hydrogen bond acceptors, **b**) hydrogen bond donors and **c**) rotatable bonds between the negative data sampled from ZINC and those sampled from the generator at different stages.

## Figure S6. Some molecules with low SA scores.


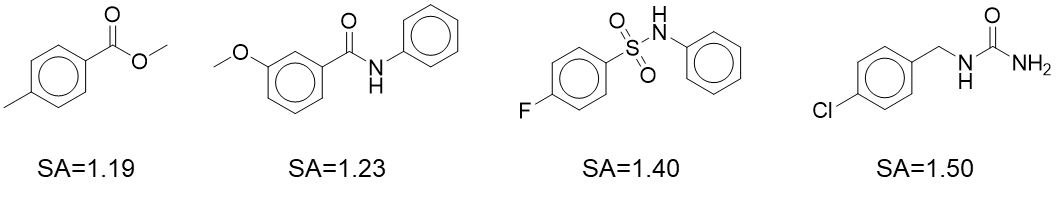


**Figure S6**. Some molecules with low SA scores. SA values can range between one (easy to synthesis) and ten (hard to synthesis).

# Tables

## Table S1, Details of the token vocabulary.

**Table S1**. The token vocabulary of MolFilterGAN.

| Token | Index | Token | Index |
| --- | --- | --- | --- |
| PAD | 0 | L | 18 |
| > | 1 | - | 19 |
| < | 2 | o | 20 |
| c | 3 | 4 | 21 |
| C | 4 | / | 22 |
| ( | 5 | [nH] | 23 |
| ) | 6 | # | 24 |
| 1 | 7 | R | 25 |
| O | 8 | [C@@] | 26 |
| 2 | 9 | \ | 27 |
| N | 10 | [C@] | 28 |
| = | 11 | 5 | 29 |
| n | 12 | [S@] | 30 |
| 3 | 13 | I | 31 |
| [C@H] | 14 | [S@@] | 32 |
| [C@@H] | 15 | [H] | 33 |
| F | 16 | 6 | 34 |
| S | 17 | P | 35 |

## Table S2, The parameters for the generator.

**Table S2**. The detail parameters for the generator of MolFilterGAN.

| Vocabulary  size | Embedding  size | LSTM | Dropout | Linear layer  (input size, output size) |
| --- | --- | --- | --- | --- |
| 36 | 128 | 3 layers and hidden size is 512 | 0.5 | (512, 36) |

## Table S3, The parameters for the discriminator.

**Table S3**. The detail parameters for the discriminator of MolFilterGAN. The vocabulary size, embedding size and dropout are the same as those of the generator.

| Kernels (In channel size,  out channel size,  sequence length, embedding dim) | Activation  function | Pooling | Concatenation (output dim) | Linear layer  (input size,  output size) |
| --- | --- | --- | --- | --- |
| (1,100,1,128), (1,200,2,128), (1,200,3,128), (1,200,4,128), (1,100,6,128), (1,100,7,128), (1,100,8,128), (1,100,9,128), (1,100,10,128), (1,160,15,128), (1,160,20,128) | Elu | Max-pooling | 1520 | (1520,1) |

## Table S4, The impact of the percentage of labeled data in positive class.

**Table S4**. The relationship between structural diversity and the percentage of labeled data in positive class.

| Percentage  (%) | Number of clusters/  Number of molecules |
| --- | --- |
| 1 | 0.99 |
| 5 | 0.94 |
| 10 | 0.90 |
| 30 | 0.78 |
| 50 | 0.71 |
| 70 | 0.66 |
